# Seroprevalence of tick-borne encephalitis virus and vaccination coverage of tick-borne encephalitis, Sweden, 2018 to 2019

**DOI:** 10.2807/1560-7917.ES.2024.29.2.2300221

**Published:** 2024-01-11

**Authors:** Bo Albinsson, Tove Hoffman, Linda Kolstad, Tomas Bergström, Gordana Bogdanovic, Anna Heydecke, Mirja Hägg, Torbjörn Kjerstadius, Ylva Lindroth, Annika Petersson, Marie Stenberg, Sirkka Vene, Patrik Ellström, Bengt Rönnberg, Åke Lundkvist

**Affiliations:** 1 Zoonosis Science Center, Department of Medical Biochemistry and Microbiology, Uppsala University, Uppsala, Sweden; 2 Laboratory of Clinical Microbiology, Uppsala University Hospital, Uppsala, Sweden; 3 Zoonosis Science Center, Department of Medical Sciences, Uppsala University, Uppsala, Sweden; 4 Department of Infectious Diseases, Institute of Biomedicine, University of Gothenburg, Gothenburg, Sweden; 5 Department of Clinical Microbiology, Karolinska University Hospital, Stockholm, Sweden; 6 Centre for Research and Development, Uppsala University, Region Gävleborg, Gävle, Sweden; 7 Laboratory Medicine, Clinical Microbiology, Central Hospital, Karlstad, Sweden; 8 Department of Laboratory Medicine, Medical Microbiology, Lund University, Skåne Laboratory Medicine, Lund, Sweden; 9 Department of Clinical Chemistry and Transfusion Medicine, Växjö Central Hospital, Växjö, Sweden; 10 Laboratory Medical Center Gotland, Visby hospital, Visby, Sweden; * These authors contributed equally to the work and share the first authorship.

**Keywords:** Tick-borne encephalitis, Sweden, seroprevalence, vaccination coverage, whole virus, non-structural protein 1

## Abstract

**Background:**

In Sweden, information on seroprevalence of tick-borne encephalitis virus (TBEV) in the population, including vaccination coverage and infection, is scattered. This is largely due to the absence of a national tick-borne encephalitis (TBE) vaccination registry, scarcity of previous serological studies and use of serological methods not distinguishing between antibodies induced by vaccination and infection. Furthermore, the number of notified TBE cases in Sweden has continued to increase in recent years despite increased vaccination.

**Aim:**

The aim was to estimate the TBEV seroprevalence in Sweden.

**Methods:**

In 2018 and 2019, 2,700 serum samples from blood donors in nine Swedish regions were analysed using a serological method that can distinguish antibodies induced by vaccination from antibodies elicited by infection. The regions were chosen to reflect differences in notified TBE incidence.

**Results:**

The overall seroprevalence varied from 9.7% (95% confidence interval (CI): 6.6–13.6%) to 64.0% (95% CI: 58.3–69.4%) between regions. The proportion of vaccinated individuals ranged from 8.7% (95% CI: 5.8–12.6) to 57.0% (95% CI: 51.2–62.6) and of infected from 1.0% (95% CI: 0.2–3.0) to 7.0% (95% CI: 4.5–10.7). Thus, more than 160,000 and 1,600,000 individuals could have been infected by TBEV and vaccinated against TBE, respectively. The mean manifestation index was 3.1%.

**Conclusion:**

A difference was observed between low- and high-incidence TBE regions, on the overall TBEV seroprevalence and when separated into vaccinated and infected individuals. The estimated incidence and manifestation index argue that a large proportion of TBEV infections are not diagnosed.

**Key public health message**

**What did you want to address in this study?**

Tick-borne encephalitis (TBE) is a vaccine-preventable viral disease affecting the central nervous system. Infections occur in Sweden, but little is known on how many people had a TBE virus infection or are protected from disease by antibodies after vaccination. We analysed serum samples from blood donors with a method that can distinguish between antibodies produced by vaccination and infection and compared the results with the number of notified TBE cases.

**What have we learnt from this study?**

We found that 1–7% of the Swedish blood donors had previously been infected by the virus and that there was a difference between regions with a low and high known frequency of the disease. We estimated that more than 160,000 individuals could have undergone the infection and more than 1,600,000 individuals had antibodies from vaccination against the disease. The results indicate that more individuals have had TBE virus infection than was previously estimated.

**What are the implications of your findings for public health?**

The findings may lead to an improved understanding of TBE in Sweden, both in regions with a few TBE cases and in those with many cases. The finding that more people have had TBE virus infection than was previously known can probably be generalised to other countries where TBE regularly occurs and guide public health authorities and the general public.

## Introduction

Tick-borne encephalitis virus (TBEV) is a member of the *Flavivirus* genus of the *Flaviviridae* family [1]. The virus is a health risk for humans and is prevalent in large areas of forested parts of Europe and in parts of Asia [2]. Three main TBEV subtypes have been identified: the European (TBEV-Eur), the Siberian (TBEV-Sib) and the Far Eastern (TBEV-FE) [1]. Two other subtypes, the Baikalian (TBEV-Bkl) and the Himalayan subtype (TBEV-Him), have also been described [3]. The geographical distribution of the TBEV subtypes mimics the geographical distribution of the main vectors, the sheep tick *Ixodes ricinus* for the TBEV-Eur subtype and the taiga tick *I. persulcatus* for the TBEV-Sib and the TBEV-FE subtypes, but mixing of subtypes in the two vector species occurs [4]. Although TBEV is mainly transmitted to humans via tick bites, consumption of unpasteurised milk and milk products has also been reported as a source of infection [5-7]. The reservoir hosts of the virus are the ticks themselves and small mammals (e.g. voles and mice), while larger wild mammals (e.g. roe deer (*Capreolus capreolus*)) act as maintenance hosts for ticks [8]. The 11 kilobases long TBEV genome encodes a single polyprotein representing three structural proteins, capsid (C), membrane (M), envelope (E) and seven non-structural (NS) proteins (NS1, NS2A, NS2B, NS3, NS4A, NS4B and NS5) [9].

The virus affects the central nervous system and tick-borne encephalitis (TBE) can vary from mild to severe illness. The disease typically presents as a two-phase illness. In the initial phase, individuals may experience symptoms, such as fever, fatigue, headache, muscle aches and nausea. The second phase involves the neurological system and includes symptoms of meningitis and/or encephalitis [10]. The case fatality rate in Europe is estimated to 1–2% [1,10]. Long-lasting sequelae may occur after an infection [1,10,11]. The frequency of subclinical infections is largely unknown, but they are considered to be quite common [1,12]. No specific treatment is available in Europe, although post-exposure immunoglobulins are available in Russia [1,3,12]. Effective vaccines exist and give good protection, but booster doses are needed, and vaccine breakthroughs or failures occur [13-15].

Tick-borne encephalitis is an important and growing public health problem in Europe [16-18]. There is a common European case definition which is used in various versions [10]. In 2020, the European Centre for Disease Prevention and Control (ECDC) received information of 3,817 notified cases from 24 European Union/European Economic Area (EU/EEA) countries [19]. Notification is compulsory in 19 countries and the notification rate was highest in Lithuania (24.3 cases per 100,000 inhabitants), followed by Slovenia and Czechia (8.9 cases per 100,000 inhabitants). The numbers of notified cases are increasing, and a spread to new regions and countries has been observed, which highlights the need for improved surveillance and updated vaccination recommendations [6,13,19-21].

Since 2004, notification of TBE cases has been mandatory in Sweden. As in the other parts of Europe, the number of notified cases in Sweden has increased despite increased vaccination, judged by increased number of vaccine doses sold along with a spread of TBE cases to new geographical regions [18,22]. The annual notified incidences of TBE vary largely between Swedish regions, from 0 in the north to > 10 cases per 100,000 inhabitants in the southern parts of the country. Currently, TBE is endemic in the southern part of Sweden with almost all cases reported from the southern third of the country. Vaccination against TBE is not included in the national vaccination programme in Sweden and there is no national vaccination registry for TBE, but it can be assumed that the proportion of vaccinated individuals increases over time, as the number of doses sold per year has increased, based on sales statistics from vaccine companies. However, there is a lack of information on TBEV seroprevalence and vaccination coverage of TBE in the Swedish population [23-26].

In this study, we used a serological method, a TBEV suspension multiplex immunoassay (TBEV SMIA), that can distinguish antibodies induced by vaccination from antibodies elicited by infection by simultaneously detecting antibodies directed against the whole virus (WV) and NS1 antigens [27,28]. The TBEV NS1 antigen is absent, or possibly present in minute amounts, in the TBE vaccines available in the EU/EEA area ‒ FSME-Immune (Pfizer) and Encepur (Bavarian Nordic) [29-33]. Hence, TBE-vaccinated individuals only develop detectable levels of antibodies against the WV antigen whereas individuals exposed to TBEV develop antibodies against both WV and NS1. The TBEV SMIA is currently employed as a national reference method for TBE diagnostics at the Laboratory of Clinical Microbiology, Uppsala University Hospital, Sweden.

The aim of the study was to estimate the TBEV seroprevalence in Sweden, including infection and vaccination coverage, using blood donor sera collected 2018–2019 from nine different regions in Sweden.

## Methods

### Serum samples

In 2018 and 2019, 2,700 blood donor sera were collected from nine regions of Sweden (Gotland, Gävleborg, Kronoberg, Skåne, Stockholm, Uppsala, Värmland, Västerbotten, Västra Götaland), 300 samples per region. In 2018, sera were collected from all regions except for Kronoberg where samples were collected in the beginning of 2019. The anonymised serum samples were routinely tested for blood-borne infections and collected without being selected by the medical doctor in charge of the clinical microbiological laboratory of the region. The exact donation site was unknown in regions with more than one blood donation site. Information on gender and age as well as donor residency was not available.

### Grouping of geographical regions

The regions included were grouped into three TBE incidence levels based on the mean number of notified TBE cases per 100,000 inhabitants 2004–2018: (i) absent–low (0–1 case), (ii) low–medium (1–4 cases) and (iii) high (> 4 cases). Incidence data were retrieved from the Public Health Agency of Sweden [22].

### Tick-borne encephalitis virus suspension multiplex immunoassay

A previously developed, evaluated and published Luminex-based TBEV SMIA for detection of antibodies against TBEV WV and NS1 antigens was used for the analyses [27,28]. Briefly, TBEV WV and NS1 antigens were coupled to differentially colour-marked magnetic microspheres. Serum samples diluted 1:50 were added to 96-well microtitre plates. After re-suspension of the microspheres, a vortexed and sonicated microsphere mixture was added to each well, giving a final serum dilution of 1:100. After 60 min incubation at room temperature (RT) and a washing step, biotinylated protein G was added, followed by a 30 min incubation at RT followed by a washing step and re-suspension. Streptavidin–phycoerythrin at a concentration of 2 μg/mL was then added and incubated for 15 min at RT. Finally, the mixture was washed once prior to re-suspension. The analysis was performed using a Luminex 200 instrument (Luminex Corporation, Austin, the United States) and the output data were reported as median fluorescence intensity (MFI). The assay cut-off values were 200 and 250 MFI for NS1 and WV, respectively. Samples were classified into three subgroups depending on the TBEV SMIA results: i) vaccinated (WV positive and NS1 negative), ii) infected (WV positive and NS1 positive) and iii) negative (WV negative and NS1 negative).

Due to the absence of methods capable of detecting antibodies against TBEV NS1 on the commercial market, a classical determination of the analytical performance of the TBEV SMIA is lacking. Clinical sensitivity has been calculated to 86–100%, depending on the antibody isotype and antigen, using patient samples from defined TBE cases. Clinical specificity has been calculated to 94–100%, depending on antibody isotype and antigen, using samples from a vaccine trial [27]. See Supplementary material for more detailed information.

### Calculations and statistics

Calculations of TBEV seroprevalence, including confidence intervals, were performed with R version 4.1.1 (R Core Team, 2021), using the Exact method in the package *binom* [34]. The estimated number of vaccinated and infected individuals was calculated by multiplying the population number with the percentage of vaccinated and infected in the region. To rule out children and a part of the elderly population and hereby reflect the blood donor as a group, we included the age group 15–64 years in the calculations (Data source: Statistics Sweden, www.scb.se). The manifestation index (MI), which is a ratio between notified cases of TBE and the number with the antibody pattern indicative of TBEV infection, was calculated as reported by Euringer et al. [35].

## Results

### Level of incidence of tick-borne encephalitis

The calculated and determined level of TBE incidence was absent–low in Gävleborg, Kronoberg, Skåne and Västerbotten; low–medium in Gotland, Värmland and Västra Götaland; and high in Stockholm and Uppsala, the latter with the highest mean number of notified TBE cases per 100,000 inhabitants 2004–2018 (Table 1).

**Table 1:** Annual notified incidence of tick-borne encephalitis and seroprevalence of tick-borne encephalitis virus in blood donors^a^, per region, Sweden, 2004–2019 (n = 2,700)

Region	TBE incidence^b^		Sera	TBEV seroprevalence								
				Vaccinated^d^			Infected^e^			Total		
	Rate	Level^c^		n	%	95% CI	n	%	95% CI	n	%	95% CI
Gotland	2.4	Low–medium	300	39	13.0	9.5–17.5	3	1.0	0.2–3.0	42	14.0	10.3–18.4
Gävleborg	0.7	Absent–low	300	54	18.0	13.9–22.9	3	1.0	0.2–3.0	57	19.0	14.7–23.9
Kronoberg	0.7	Absent–low	300	26	8.7	5.8–12.6	3	1.0	0.2–3.0	29	9.7	6.6–13.6
Skåne	0.6	Absent-low	300	53	17.7	13.6–22.6	4	1.3	0.4–3.6	57	19.0	14.7–23.9
Stockholm	4.8	High	300	171	57.0	51.2–62.6	21	7.0	4.5–10.7	192	64.0	58.3–69.4
Uppsala	9.1	High	300	84	28.0	23.1–33.5	9	3.0	1.2–5.4	93	31.0	25.8–36.6
Värmland	1.5	Low–medium	300	97	32.3	27.1–38.0	9	3.0	1.5–5.8	106	35.3	29.9–41.0
Västerbotten	0.1	Absent–low	300	34	11.3	8.1–15.6	3	1.0	0.2–3.0	37	12.3	8.8–16.6
Västra Götaland	1.6	Low–medium	300	120	40.0	34.5–45.8	9	3.0	1.5–5.8	129	43.0	37.3–48.8
Total			2,700	678	25.1	23.5–26.8	64	2.4	1.8–3.0	742	27.5	25.8–29.2

CI: confidence interval; NS1: non-structural protein 1; TBE: tick-borne encephalitis; TBEV: tick-borne encephalitis virus; WV: whole virus.

^a^ Serum samples from blood donors in Gotland, Gävleborg, Skåne, Uppsala, Värmland, Västerbotten and Västra Götaland were taken 2018 and those from Kronoberg in 2019.

^b^ Annual notifications per 100,000 inhabitants 2004–2018. Data source: Public Health Agency of Sweden (https://www.folkhalsomyndigheten.se/the-public-health-agency-of-sweden/).

^c^ Absent–low: 0–1 case/100,000 inhabitants; low–medium: 1–4 cases/100,000 inhabitants; high: > 4 cases/100,000 inhabitants.

^d^ Individuals were defined as vaccinated if antibodies were detected against TBEV WV but not against NS1.

^e^ Individuals were defined as infected if antibodies were detected against TBEV WV and NS1.

### Seroprevalence of tick-borne encephalitis virus

The overall seroprevalence among all blood donors was 27.5% and varied from 9.7 to 64.0% between the regions and the proportions of vaccinated and infected individuals from 8.7 to 57.0% and 1.0 to 7.0%, respectively (Table 1). Blood donors in Stockholm, Västra Götaland, Värmland and Uppsala region had the highest overall seroprevalence (64.0%, 43.0%, 35.3% and 31.0%), vaccination coverage (57.0%, 40.0%, 32.3% and 28.0%) and proportion of infected individuals (7.0%, 3.0%, 3.0% and 3.0%). The proportions of TBE vaccinated, infected and seronegative (unvaccinated/uninfected) blood donors per region and the geographical location of the regions included are presented in the Figure. Seven serum samples (0.3%) tested positive for only NS1 (WV negative and NS1 positive) in two separate runs.

**Figure:**
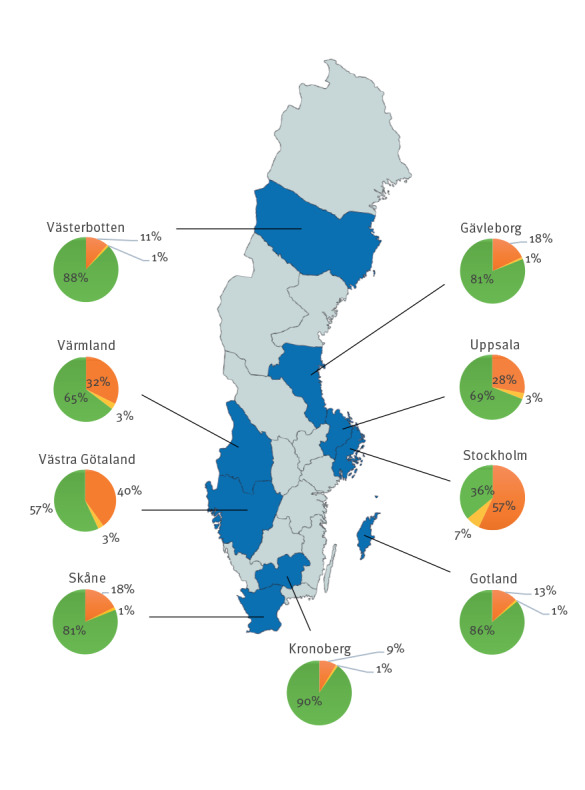
Estimated proportion of tick-borne encephalitis vaccinated, tick-borne encephalitis virus infected and seronegative blood donors, per region, Sweden, 2018–2019 (n = 2,700)

Proportions of blood donors vaccinated depicted in orange, infected in yellow and seronegative (unvaccinated or uninfected) in green per investigated region in blue. In total, 300 blood donor sera collected in 2018 (Gotland, Gävleborg, Skåne, Uppsala, Värmland, Västerbotten and Västra Götaland) or 2019 (Kronoberg) were analysed per region.

The map was created with MapChart (https://www.mapchart.net/).

### Estimated total number of individuals vaccinated against tick-borne encephalitis and infected by tick-borne encephalitis virus

In 2018 and 2019, an estimated 1,644,100 individuals aged 15–64 years were vaccinated against TBE and 168,000 had been infected by TBEV in the studied regions when recalculated from the blood donor seroprevalence data (Table 2). The regions with the highest population sizes (Stockholm, Västra Götaland, Skåne and Uppsala) had the highest estimated numbers of vaccinated and infected individuals, as well as the highest numbers of notified TBE cases 2004–2018.

**Table 2:** Estimated number of individuals aged 15–64 years infected by tick-borne encephalitis virus, vaccinated against tick-borne encephalitis and notified number of tick-borne encephalitis cases, per region, Sweden, 2004–2019

Region	Population^a^	Estimated TBE vaccinated^b^		Estimated TBEV infected^b^		TBE notifications 2004–2018^c^	
		n	95% CI	n	95% CI	n	Incidence
Gotland	35,046	4,600	3,300–6,100	400	100–1,100	21	2.5
Gävleborg	170,671	30,700	23,700–39,100	1,700	500–5,300	28	0.7
Kronoberg	121,274	10,600	7,000–15,300	1,200	400–3,800	21	0.7
Skåne	848,053	150,100	115,300–191,700	11,000	3,400–30,500	107	0.6
Stockholm	1,534,225	874,500	785,500–960,400	107,400	69,000–164,200	1,521	4.8
Uppsala	239,927	67,000	55,300–80,200	7,200	2,900–12,900	469	9.1
Värmland	168,738	54,500	45,700–64,100	5,100	2,500–9,800	61	1.5
Västerbotten	167,765	21,800	13,600–26,200	1,700	500–5,200	5	0.2
Västra Götaland	1,075,801	430,300	371,200–492,700	32,300	16,100–62,400	394	1.6
Total	4,360,870	1,644,100		168,000		2,627	

CI: confidence interval; TBE: tick-borne encephalitis; TBEV: tick-borne encephalitis virus.

^a^ Age group 15–64 years. Source: Statistics Sweden (www.scb.se), 31 December 2018.

^b^ Serum samples from blood donors in Gotland, Gävleborg, Skåne, Uppsala, Värmland, Västerbotten and Västra Götaland were taken in 2018 and those from Kronoberg in 2019.

^c^ All age groups. Source: Public Health Agency of Sweden (https://www.folkhalsomyndigheten.se/the-public-health-agency-of-sweden/).

### Calculated manifestation index

The calculated manifestation index (MI) ranged from 0.4 to 8.7% between the studied regions (Table 3). Uppsala had the highest MI and Västerbotten the lowest. The mean MI was 3.1%.

**Table 3:** Calculated manifestation indices of tick-borne encephalitis, per region, Sweden, 2004–2019

Region	Notified TBE case incidence^a^	TBEV infection incidence^b^	TBE MI (%)
Gotland	4.0	50.0	8.0
Gävleborg	1.1	50.0	2.2
Kronoberg	1.2	50.0	2.3
Skåne	0.8	66.7	1.3
Stockholm	6.6	350.0	1.9
Uppsala	13.1	150.0	8.7
Värmland	2.4	150.0	1.6
Västerbotten	0.2	50.0	0.4
Västra Götaland	2.4	150.0	1.6
Mean	3.5	118.5	3.1

MI: manifestation index; NS1: non-structural protein 1; TBE: tick-borne encephalitis; TBEV: tick-borne encephalitis virus; WV: whole virus.

^a^ Annual notifications per 100,000 inhabitants 2004–2018. Data source: Public Health Agency of Sweden (https://www.folkhalsomyndigheten.se/the-public-health-agency-of-sweden/).

^b^ Infected (WV positive and NS1 positive) per 100,000 inhabitants and year; estimated duration of TBEV NS1 IgG: 20 years.

## Discussion

We investigated the TBEV seroprevalence among blood donors indicative of previous infection and vaccination in nine Swedish regions. The overall TBEV seroprevalence during the study period was 27.5%, ranging from 9.7% to 64.0% between the regions. Previous studies in Sweden have included fewer regions, which complicates comparisons with other studies [23-26].

We used the mean number of notified TBE cases per 100,000 inhabitants from 2004 to 2018 to categorise the incidence levels. Since the number of notified TBE cases has increased over time and cases are notified in new areas, our calculations do not accurately reflect the most recent situation. For instance, in 2004, Värmland and Västra Götaland had a TBE incidence of less than one case per 100,000 inhabitants. In 2018, the TBE incidence had increased to 4.3 cases per 100,000 inhabitants in Värmland and to 3.5 per 100,000 inhabitants in Västra Götaland.

According to the World Health Organization (WHO), the TBE incidence in an area is high if more than 5 cases per 100,000 inhabitants are annually notified and moderate or low if fewer than 5 cases per 100,000 inhabitants are notified [36]. We used a three-point TBE incidence scale (absent–low, low–medium and high) to compare our results on TBEV seroprevalence with notified TBE incidence. A difference was seen in the seroprevalence when regions of low TBE incidence were compared with those of high incidence, both concerning the overall seroprevalence and when divided into subgroups of vaccination and infection. Gotland and Uppsala were the two exceptions in the study. For several years, Uppsala and Stockholm regions have had a high TBE incidence, and Uppsala for several consecutive years [22]. In our study, Uppsala region had a lower seroprevalence compared with Stockholm, but similar to Värmland and Västra Götaland regions with a low–medium incidence. This finding was unexpected, since Uppsala has been a high incidence region for many years and usually with a higher incidence than Stockholm. This high incidence in Uppsala region is likely due to hot spot areas of TBEV, a high abundance of ticks in the vicinity of lakes, watercourses and other tick habitats and many inhabitants living in the countryside. The lower TBEV seroprevalence in the Uppsala region in our study could be explained by some serum samples originating from individuals residing in an urban area in the Gävleborg region, which has an absent–low TBE incidence. We were not aware of this during sample collection and serological analyses. Gotland is an island and has a small population, which leads to relatively high variation in incidence between years, as isolated cases can greatly impact the incidence rate. For the other regions, including Stockholm, the TBEV seroprevalences of this study were congruent with notified incidences.

By measuring antibodies against NS1 we estimated that 2.4% of the blood donors had a past TBEV infection. The NS1 IgG seroprevalence ranged from 1.0 to 7.0% and was highest in the Stockholm region with a high TBE incidence. The latter infection seroprevalence is in line with a study by Euringer et al., a TBEV NS1 IgG seroprevalence of 5.6% in blood donors from a highly endemic district in south-western Germany measured in 2021 [35]. Information about the persistence of TBEV NS1 specific IgG is limited. In one patient, anti-TBEV NS1 IgG could be detected 28 years after confirmed TBE [33], indicating long-term immunity. We estimated the total number of infected and vaccinated individuals per region by extrapolating the seroprevalence results to the population in the studied regions. According to our estimations, more than 160,000 individuals could have been infected by TBEV and 1,600,000 individuals could be TBE vaccinated, in the study regions and in the age group 15–64 years. Assuming anti-TBEV NS1 IgG (and anti-TBEV WV IgG) can be detected for approximately 20 years after infection, it is probable that this seroprevalence has been accumulated during the last two decades. Although a larger sample size would provide more accurate estimates, we consider that these results give an important indication of the true prevalence, given the scarce knowledge on TBEV infections in Sweden. A notable part of TBEV infections is assumed mild or subclinical, however, our results indicate that a large proportion of TBEV infections are not diagnosed and therefore not notified. Reasons for this may be subclinical infections, mild and rapidly transient symptoms or delay of diagnosis, i.e. TBEV infection is not considered so TBEV testing is not performed. Information on the estimated number of vaccinated individuals is of value although our data do not contain detailed information on vaccination, such as the number of given vaccine doses or the immunity of the blood donors.

We included nine Swedish regions with notified TBE incidences ranging from absent and low to high. The mean MI was 3.1%, which is in line with the MI from a highly TBE endemic district in Germany (2%) [35]. Thus, most TBEV infections (96.9%) are not diagnosed and thereby not notified in Sweden. For future comparisons of European TBE manifestation indices of different countries, a harmonisation of the TBE case definition in the EU is needed [10]. Comparing the MI in a region over time could be a way to detect a potential shift to another subtype of TBEV. For example, a more pathogenic subtype or variant could result in a higher MI as a greater proportion of the infected individuals would be likely to seek medical care and be diagnosed. This would require more continuous monitoring of seroprevalence. In Sweden, the notified TBE incidence has increased since the disease became notifiable in 2004, with some fluctuation between the years [22], even though there are good vaccines available on the market in Sweden since the 1980s and vaccination campaigns are common in high-risk areas. A top record of TBE cases in Sweden was noted in 2021 – one year into the coronavirus disease 2019 (COVID-19) pandemic, with a doubling of cases from the year before. The number of reported cases will be even higher in the year 2023 [22]. The increasing TBE incidence and the high number of notified TBE cases in 2021 are likely a result of several factors, such as an increasing tick and rodent abundance, climatic conditions favouring virus replication and tick phenology (the timing of host-seeking activity), increased awareness by doctors leading to increased diagnosis, a decrease in given vaccine doses during 2021 [37] and changes in human behaviour leading to increased contact between humans and infected ticks, i.e. more individuals spending more time in the same habitat as ticks (e.g. increased outdoor activities during the COVID-19 pandemic due to contact and travel restrictions) [22,38]. We would likely have seen more TBE cases without vaccination, which stresses the importance of immunising individuals living, visiting and working in TBE risk areas, as well as the need for a national vaccination registry in Sweden. A national registry would not only help individuals to follow the vaccination recommendations but also to vaccinate in time, i.e. before the start of the tick season. Unfortunately, it cannot be ruled out that the cost of vaccination could have a negative influence on the willingness to vaccinate against TBE in Sweden as there is no government subsidy. However, some Swedish regions (Södermanland, Uppsala and Östergötland) have introduced free vaccinations for children aged 3–18 years and more regions seem to follow.

The geographical distribution and seasonality of TBE cases correspond to the geographical distribution and seasonality of ticks. In the northern hemisphere, tick habitats are connected to water. Most TBE cases in Sweden have thus been reported from areas around Lake Mälaren and the Stockholm archipelago, but a northern and western spread has been observed, including areas around Lake Vänern and north of Gothenburg [22]. The ability of *Ixodes* ticks to spread to new areas is highly influenced by the presence of hosts, temperature and precipitation or humidity [38,39]. Additionally, the phenology of ticks is temperature-dependent [39]. Most TBE cases are reported during the tick season: in Sweden between July and September, but cases are reported also in January and December [22]. Climate change has rapidly changed the distribution of *Ixodes* ticks and the pathogens they transmit. In Sweden, this has resulted in an expanding geographical range and longer host-seeking periods for *I. ricinus* [38,40], explaining why TBE cases are reported from new regions but also during the winter season. Since the epidemiology of TBE is changing and ticks are expanding their geographical range in Sweden [41], sentinel monitoring using serological examination of TBEV in wildlife and bulk tank milk [42], the latter allowing easier sampling, should be performed nationally. All regions that report detection of TBEV in wildlife and bulk milk should be classified as a potential risk area for TBE and recommend TBE vaccination to everyone at risk of getting a tick bite in the region. At present, it is up to each region to issue vaccination recommendations based on their local situation.

Sera from blood donors were used in this study, so a generalisation of the results to the entire population should be made with caution. Blood donors are probably healthier and have a more active lifestyle than others, including outdoor activities where exposure to TBEV can be expected [43]. Blood donors are adults, aged 18–65 years, so children and elderly individuals are not included. Blood donors may have a more positive attitude to vaccinations, including TBE vaccination. Furthermore, TBE is more commonly diagnosed among men in Sweden [22] and Swedish men are slightly over-represented among blood donors in the age group 25–64 years [44]. Information on age and gender as well as residency was not available for the blood donors, which is a limitation of this study. Some regions have more than one blood donation site. The serum samples in our study could originate from different sites of the region, making it impossible to differentiate the results beyond the region level. Flaviviruses can cross-react, especially for IgG. False positive IgG-reactivity against, for example, dengue or yellow fever may occur, but we assess the risk as low and limited to those who may have been exposed during travel or vaccination, as no other flaviviruses than TBEV are endemic in Sweden. There is an ongoing discussion about the possible presence of NS1 protein in the TBE vaccines used in Europe [29-32]. As most studies have either not detected the NS1 protein or detected minute amounts in the vaccine preparations, we assume that any potential NS1 antibodies in samples from individuals vaccinated would be present in amounts below the detection limit of the method used. Of the serum samples included in this study, 0.3% tested positive for only anti-TBEV NS1 antibodies. We regarded these samples as unspecific.

## Conclusion

In conclusion, we present seroprevalence results of TBEV in samples from blood donors from nine Swedish regions. In general, there was a difference in the seroprevalence when regions of low TBE incidence were compared with those of high incidence, both concerning the overall seroprevalence and when divided into subgroups of vaccination and infection. The results also indicate that a large proportion of TBEV infections in Sweden are not diagnosed and therefore not notified as cases, possibly because of mild symptoms or subclinical disease.

## Supplementary Data

172000 Supplementary Material
