# Supplementary material for: Seroprevalence of tick-borne encephalitis virus and vaccination coverage of tick-borne encephalitis, Sweden, 2018 to 2019
Source: Euro Surveill. 2024 Jan 11;29(2):2300221. doi: 10.2807/1560-7917.ES.2024.29.2.2300221 (PMC10785208; doi:10.2807/1560-7917.ES.2024.29.2.2300221)
Supplement: Supplementary Material [file 23-00221_ALBINSSON_Supplement.pdf]

Clinical performance of the TBEV SMIA

Conventional analysis of the analytical performance of the tick-borne encephalitis virus suspension multiplex immunoassay (TBEV SMIA) has not been performed, since no commercial method for detecting antibodies against TBEV non-structural protein 1 (NS1) is available on the market, to the best of our knowledge. Consequently, no direct analytical comparisons or performance calculations can be made.

Clinical sensitivity:
However, in our previous publication (Albinsson et al, 2018), serum samples from 50 clinical tick-borne encephalitis (TBE) cases were employed to validate the method. The results indicated that the TBEV SMIA exhibited a clinical sensitivity of 100% (50/50) for both whole virus (WV) IgG and IgM, 86% (43/50) for non-structural protein 1 (NS1) IgG, and 92% (46/50) for NS1 IgM. In the same study, serum samples from 50 TBEV vaccinees were examined. Ninety-four percent (47/50) developed antibodies against WV during the study period.

Clinical specificity:
Clinical specificity, represented by the number of samples containing antibodies against WV IgM, NS1 IgG, and NS1 IgM, was 94% (3/50), 96% (2/50), and 100% (0/50) respectively. We would like to note that it cannot be ruled out that some vaccinees might have been exposed to TBEV during the study period of 3-4 years, potentially leading to the development of NS1 IgG antibodies, for example.

Reference:
Albinsson B, Vene S, Rombo L, Blomberg J, Lundkvist Å, Rönnberg B. Distinction between serological responses following tick-borne encephalitis virus (TBEV) infection vs vaccination, Sweden 2017. Eurosurveillance. 2018 Jan 18 23(3).

Disclaimer:
This supplementary material is hosted by Eurosurveillance as supporting information alongside the article Seroprevalence of tick-borne encephalitis virus and vaccination coverage of tick-borne encephalitis, Sweden, 2018 to 2019, on behalf of the authors, who remain responsible for the accuracy and appropriateness of the content. The same standards for ethics, copyright, attributions and permissions as for the article apply. Supplements are not edited by Eurosurveillance and the journal is not responsible for the maintenance of any links or email addresses provided therein.
